# Supplementary material for: SyNDI: synchronous network data integration framework
Source: BMC Bioinformatics. 2018 Nov 6;19:403. doi: 10.1186/s12859-018-2426-5 (PMC6219086; doi:10.1186/s12859-018-2426-5)
Supplement: Supplementary file 5 — Files of the example presented in Additional file 4. This additional file is a zip package that contains all files used in Additional file 4. Detailed descriptions of each of these files are presented in a README file included in this zip package. (GZ 30411 kb) [file 12859_2018_2426_MOESM5_ESM.gz › SyNDITools/Meme2Fimo/meme_4.9.0_4.tar.gz!/meme_4.9.0/doc/jaspar2meme.html]

jaspar2meme


# jaspar2meme

**Usage:** `jaspar2meme [options] <Jaspar
directory>`

**Description:**

Convert a directory of JASPAR files into a MEME version 4 formatted file
suitable for use with MEME Suite programs.

**Input:**

`<Jaspar directory>` - a directory containing one or
more JASPAR motif files. The possible formats are:

- A JASPAR '.sites' file describes a motif in terms of a multiple
  alignment of sites. It contains a multiple alignment in modified FASTA
  format. Only capitalized sequence letters are part of the alignment.
- A JASPAR count file ('.pfm') contains a count matrix where the
  rows correspond to A, C, G and T, respectively.
- A CM count file ('.cm') prefixes the rows with 'A| ', 'C| ', 'G|
  ' and 'T| '.

A probability matrix and optionally a log-odds matrix is output for each
motif file. The probability matrix is computed using
pseudo-counts consisting of the background frequency (see -bg, below)
multiplied by the total pseudocounts (see -pseudo, below).

**Options:**

-pfm
:   read JASPAR count files (.pfm); default: site files (.sites)

-cm
:   read count file with line labels 'A|' etc. (.cm); default: site files (.sites)

-numbers
:   use numbers instead of strings as motif names

-strands 1|2
:   print '+ -' '+' on the MEME strand line; default: 2 (prints '+ -')

-bg <bfile>
:   file with background frequencies in MEME -bfile format; default: uniform frequencies

-pseudo <A>
:   add <A> times background frequency to each count when computing letter frequencies default: 0

-logodds
:   print log-odds matrix as well as frequency matrix; default: frequency matrix only

-url <website>
:   website for the motif; The motif name is substituted for MOTIF\_NAME;

**Output:**

Writes MEME format to standard output.

**Sample Input:**

*.pfm format (counts):*

```
 0  3 79 40 66 48 65 11 65  0
94 75  4  3  1  2  5  2  3  3
 1  0  3  4  1  0  5  3 28 88
 2 19 11 50 29 47 22 81  1  6
```

*.cm format (counts):*

```
A|  0  3 79 40 66 48 65 11 65  0
C| 94 75  4  3  1  2  5  2  3  3
G|  1  0  3  4  1  0  5  3 28 88
T|  2 19 11 50 29 47 22 81  1  6
```

*.sites format (motif sites):*

```
>MA0024 E2F     1
aTTTGGCGC
>MA0024 E2F     2
TTTGGCGC
>MA0024 E2F     3
TTTGGCGC
>MA0024 E2F     4
TTTGGCGC
>MA0024 E2F     5
TTTCGCGC
>MA0024 E2F     6
TTTCGCGC
>MA0024 E2F     7
TTTCGCGC
>MA0024 E2F     8
TTTGCCGC
>MA0024 E2F     9
TTTCCCGC
>MA0024 E2F     10
TTTGGCGG

A  [ 0  0  0  0  0  0  0  0 ]
C  [ 0  0  0  4  2 10  0  9 ]
G  [ 0  0  0  6  8  0 10  1 ]
T  [10 10 10  0  0  0  0  0 ]
```
